# Impact of recent and future climate change on vector‐borne diseases

**DOI:** 10.1111/nyas.13950

**Published:** 2018-08-18

**Authors:** Cyril Caminade, K. Marie McIntyre, Anne E. Jones

**Affiliations:** ^1^ Department of Epidemiology and Population Health, Institute of Infection and Global Health University of Liverpool Liverpool UK; ^2^ NIHR Health Protection Research Unit in Emerging and Zoonotic Infections Liverpool UK; ^3^ Department of Mathematical Sciences University of Liverpool Liverpool UK

**Keywords:** climate change, vector‐borne disease, water‐borne disease, public health, emerging disease

## Abstract

Climate change is one of the greatest threats to human health in the 21st century. Climate directly impacts health through climatic extremes, air quality, sea‐level rise, and multifaceted influences on food production systems and water resources. Climate also affects infectious diseases, which have played a significant role in human history, impacting the rise and fall of civilizations and facilitating the conquest of new territories. Our review highlights significant regional changes in vector and pathogen distribution reported in temperate, peri‐Arctic, Arctic, and tropical highland regions during recent decades, changes that have been anticipated by scientists worldwide. Further future changes are likely if we fail to mitigate and adapt to climate change. Many key factors affect the spread and severity of human diseases, including mobility of people, animals, and goods; control measures in place; availability of effective drugs; quality of public health services; human behavior; and political stability and conflicts. With drug and insecticide resistance on the rise, significant funding and research efforts must to be maintained to continue the battle against existing and emerging diseases, particularly those that are vector borne.

## Introduction

Climate change is considered one of the greatest threats to human health by the World Health Organization. The rate of global warming which has occurred during recent decades has been unprecedented over the past millennium,^1^, ^2^ and there is consensus in the scientific community that the cause is increasing anthropogenic emissions of greenhouse gases.^2^ Climate change directly impacts health through long‐term changes in rainfall and temperature, climatic extremes (heatwaves, hurricanes, and flash floods), air quality, sea‐level rise in lowland coastal regions, and multifaceted influences on food production systems and water resources.^3^ Since 2016, the *Lancet* countdown initiative has been tracking progress on health and climate change issues related to implementation of the Paris climate agreement,^4^, ^5^ providing a broad overview of climate change impacts on health. In this review, we focus specifically on the impact of climate change on infectious diseases. This impact is likely to be significant; a recent systematic review of European human and domestic animal pathogens suggested that nearly two‐thirds were climate sensitive, many to more than one climate driver.^6^

Infectious diseases of humans and animals have played a significant role in history. Plague outbreaks in Rome (2nd century BCE) and Athens (5th century BCE) were reported in biblical records, and the Black Death epidemic which struck Europe during the 14th century wiped out between a third and a half of the European population.^7^ Subsequently, conquests of the Americas, Australia, and South Africa by Europeans were facilitated by importation of germs such as measles and smallpox that decimated fully susceptible indigenous populations.^8^ More recently, in 2014–2016, the largest yet observed Ebola outbreak devastated vulnerable populations in Sierra Leone, Liberia, and Guinea.^9^ This was followed by the Zika virus (ZIKV) epidemic affecting Latin America, the Carribean, parts of Southeast Asia, and Africa in 2015–2016. Over the summer of 2017, very moderate autochthonous transmission of ZIKV also took place in southern Florida and Texas, in the United States, a wealthy country.

The negative impact of infectious diseases on health and well‐being is intrinsically linked to a combination of multiple stressors or drivers such as poor sanitation, access to clean water and food, the quality of public health services, political instability and conflict, drug resistance, and animal and/or human population movements.^10^ How we shape and adapt to the environment, through our impact on land use (deforestation/afforestation and agricultural activities), the building of artificial water bodies or dams,^11^ and the measures undertaken to control infectious diseases such as vaccine and drug development, insecticide spraying, distribution of impregnated bed nets, and development of rapid diagnostic tests, are also critical factors affecting infectious disease transmission. Climate has a direct impact on the dynamics of a subset of infectious diseases, including vector‐borne diseases (VBDs), some water‐borne diseases such as cholera, and other soil‐borne and food‐borne pathogens.^6^ Climate also has multiple indirect effects through socioeconomic factors; as one example, flooding can hamper disease control measures in place, including vector control.^12^

Infectious VBDs are mainly transmitted by arthropod vectors, which are particularly sensitive to changes in climate, for a number of reasons. Arthropods are ectothermic, with their internal temperature regulated by external environmental conditions. Their larval development stage generally requires the presence of bodies of water and/or specific humidity conditions. Vector biting rates tend to increase with temperature up to an upper threshold, after which they decrease.^13^ The development and replication of pathogens transmitted within vectors (the extrinsic incubation period or EIP) or in the environment also occurs faster at high temperatures.^14^ Furthermore, vector development and survival is significantly affected by temperature conditions.^15^ The entomological parameters affected by rainfall and temperature can be summarized using the maximum daily reproductive rate of the disease: the vectorial capacity.^16^ The optimal temperature range for disease transmission varies depending upon the vector–pathogen combination being studied; however, vectorial capacities of the most harmful tropical VBDs consistently peak at relatively high temperatures.^17^

The evidence suggests that future climate change, if not mitigated, will very likely impact the length of the transmission season and the geographical range of a significant proportion of infectious diseases.^18^ On a broader scale, climate change will reshuffle the geographical distribution of animal species, and one of the most prominent illustrations of this is an image of a starving polar bear, released by the National Geographic Society in December 2017. The direct impact of climate change on habitat, and therefore ecosystem change, combined with increasing anthropogenic pressure on the natural environment, is severely affecting biodiversity, further impacting the emergence and transmission of infectious diseases.^19^ An important point to emphasize is that of attribution and detection: how can recent spatiotemporal changes in infectious diseases be attributed, wholly or in part, to long‐term anthropogenic climate change? This is a complicated question to answer, hindered by the lack of good quality health and climate datasets over long time periods, by the various nonclimatic factors at play and by the influence of natural climate variability modes that are now occurring in a warmer background, such as the crucial El Niño Southern Oscillation. The latter issue led to controversy over the attribution of climate change effects on recent malaria changes observed in the East African highlands.^20^, ^21^, ^22^, ^23^, ^24^, ^25^, ^26^ However, there is clear evidence that climate change has already affected the latitudinal and altitudinal ranges of avian malaria in wild birds.^27^, ^28^ The health of wild animals, particularly birds, is assumed to be a better indicator of early climate change effects because very little or no control measures are undertaken to protect them.^29^, ^30^ VBDs seriously affect the health of domestic animals and livestock (e.g., trypanosomiasis, Rift Valley Fever, and bluetongue), and consequently, climate change will also indirectly affect our health through its multifaceted impacts on food security, including livestock and plant crops.

There is a need to pragmatically estimate and discuss the importance of climate with respect to other critical factors affecting the spatiotemporal dynamics of infectious diseases. In this review, we discuss recent trends and advances in our understanding of the impact of recent and future climate change on VBD dynamics. We mainly focus on VBDs, as they are expected to be the most climate‐sensitive subset of all infectious diseases and have sensitivity to the greatest number of climate drivers.^6^ As quantitative detection and attribution of climate change impacts is impossible for most infectious diseases,^31^ we highlight recently observed trends in temperate, Arctic, and tropical‐altitude regions which have already experienced significant changes in climate, and for which one would consequently expect some evidence of early climate change impacts on VBD burden. We also discuss progress in state‐of‐the‐art future risk scenarios for VBDs, methodological issues, and the relevance of this research to policy makers and governmental health agencies.

## Recent studies

The global burden of infectious diseases has significantly declined over past decades, thanks to the development of modern medicine, the combination of poverty alleviation and socioeconomic development, and the deployment of more effective intervention and control measures.^32^ However, improvement at a global scale masks large changes occurring regionally. From 1990 to 2012, the frequency of academic studies referencing a disease‐climate link has nearly doubled.^33^ In the following, we will discuss important emerging examples of VBDs affecting humans and animals.

### Malaria

Human malaria is caused by five species of plasmodium parasites and is transmitted by female *Anopheles* mosquitoes. The tropical form, *Plasmodium falciparum*, causes the most severe clinical form of malaria and is widespread in the tropics and sub‐Saharan Africa, causing about 90% of global malaria cases.^34^ The more temperate form, *Plasmodium vivax*, used to be prevalent in Europe, but control measures such as drainage of marshes and spraying of dichlorodiphenyltrichloroethane (DDT) led to its disappearance following World War II. Overall, *Anopheles* mosquitoes need adequate rainfall to create breeding sites that will not dry up or wash away over a 9–12 day period. Replication of the parasite within the mosquito vector requires a minimum air temperature of about 15–16 °C for *P. vivax* and 19–20 °C for *P. falciparum*.^35^, ^36^ Increased temperatures that are close to the upper limit for vector and pathogen survival (roughly about 35–37 °C) tend to reduce transmission, while increased variation in daily temperatures near the lower limit tends to increase transmission.^35^, ^37^, ^38^ Precipitation creates adequate vector breeding sites, and the relationship between temperature, rainfall, and malaria is also highly vector‐specific.^24^, ^39^

Economic development and the implementation of stringent control and intervention measures have driven a large global decrease in the spatial distribution and endemicity of the disease over the past century. This is despite rising global temperatures,^40^, ^41^ thanks to significant funding efforts to control and reduce malaria burden. Current malaria interventions have the ultimate goal of malaria elimination, but are unlikely to be capable of eradicating the disease completely in all regions of Africa, even in current climate conditions.^42^ The number of reported human infections with *P. falciparum* is increasing in tropical highland regions across the globe, for example, in Eastern Africa,^24^, ^25^, ^43^, ^44^, ^45^, ^46^ Nepal,^47^, ^48^, ^49^, ^50^ and Colombia.^43^ Competent malaria vectors have also recently been found at higher altitudes.^47^, ^49^, ^51^, ^52^, ^53^, ^54^This has serious implications for indigenous highland human populations that usually lack protective immunity and are more vulnerable to severe malaria morbidity and mortality. The extraordinary biological complexity of the malaria parasite has hindered the development of effective vaccines, even though it is well known that transmission‐blocking antiparasite immune responses exist. RTS,S/AS01 is the most advanced malaria vaccine candidate currently in development, and is due to be rolled out in trials in three African countries in 2018.^55^

Other malaria parasites also affect animals, including reptiles, birds^27^, ^28^ (with infections recently reported as far north as Alaska^56^), and some mammals. Occasionally, humans are infected by *Plasmodium* species that normally infect animals (e.g., *Plasmodium knowlesi*, usually transmitted in small primates).^57^ Fully susceptible penguins have been severely impacted by avian malaria in UK zoos in 2016. This might be related to both conducive environmental conditions for both the vector and parasite, and the importation of parasites in migratory birds.

Several studies have estimated the potential impact of climate change on the distribution and severity of *P. falciparum* human malaria at global and regional scales using malaria models driven by climate model simulations.^58^, ^59^, ^60^, ^61^, ^62^, ^63^, ^64^ Overall, future climate conditions in these simulations are increasingly suitable for malaria transmission in tropical highland regions, in particular the East African highlands; the malaria epidemic fringe is generally predicted to shift southward in sub‐Saharan Africa; and temperature conditions might become unbearable for the mosquito vectors in the warmer tropical plains of the Sahel by the end of the 21st century. Most of these studies only estimate the impact of climate on malaria without accounting for important socioeconomic factors;^40^, ^62^, ^65^ economic development and intervention might offset and counteract climate‐induced trends.^66^

Even if future climate conditions become suitable for malaria transmission in Europe, temperate Asia, and the United States (as in the past),^67^ a large epidemic is very unlikely to happen in these regions given the availability of antimalarial drugs and the standard of public health services. However, local transmission of *P. vivax* occurred in Greece in 2009–2010 in association with large cuts in government spending due to the economic crisis, human migration, and the heatwave that struck southeastern Europe.^68^ Very limited autochthonous transmission of *P. vivax* in Spain was recently reported,^69^ and a few puzzling, very likely nosocomial, *P. falciparum* infections were also reported in Italy in 2017.^70^ This is a major warning for our health services and decision makers given recent economic climate and austerity measures.^71^ A dramatic re‐emergence of malaria cases since 2000 has been observed in the temperate Anhui province of China, prior to which there was very low‐level endemicity. This sudden increase is related to climatic conditions, with rainfall strongly associated with malaria transmission.^72^ Interestingly, clinicians emphasized that they expect climate change to affect the burden of infectious diseases in the area, despite adequate hospital infrastructure to tackle emerging infectious diseases.^73^

The most worrying risk factor affecting malaria is that mosquitoes and parasites are developing resistance to the weapons we use to fight them. Vector resistance to insecticide (DDT), combined with parasite resistance to antimalarial drugs (chloroquine mainly and artemisinin to a lesser extent), is on the rise.^74^ Drug and insecticide resistance might counteract the amazing progress achieved by malaria control measures over the past 15 years. Additionally, most malaria resurgences over the 20th century were related to weakening of malaria control measures; thus, there is an urgent need to deliver practical solutions to financial and operational threats to sustain recent successful malaria control programs.^75^, ^76^ Sadly, there were 216 million cases of malaria worldwide in 2016, an increase of five million compared with 2015. In some countries and regions, particularly in sub‐Saharan Africa, malaria is on the rise again.^34^

### Important arboviruses: dengue, chikungunya, yellow fever, Zika, and West Nile

The arboviruses dengue, Zika, yellow fever, and chikungunya are transmitted by *Aedes* mosquito species; *Aedes albopictus* (the Asian tiger mosquito) and *Aedes aegypti* (the yellow fever mosquito) are thought to be their main competent vectors. Dengue is the most rapidly spreading mosquito‐borne disease, with a 30‐fold increase in global incidence over the past 50 years^77^ and an estimated 100–390 million dengue infections reported worldwide each year.^78^ These vectors have spread into new areas due to globalization and international trade, for example, importation of used tires and plants from Asia.^79^, ^80^, ^81^, ^82^ This group of diseases poses serious concerns for public health services, given the heavy burden they still cause in Asia and South America,^78^ the large potential of *Aedes* mosquitoes to transmit viruses in urban settings,^83^, ^84^ and the risk of autochthonous transmission of diseases by returning infected travelers in vector‐endemic regions.^85^, ^86^

No fully effective vaccine has yet been developed for these diseases^87^, ^88^ but such work is in progress.^89^, ^90^ Recent trends are worrying, with outbreaks of dengue reported in the United States,^91^ Madeira in 2012,^92^ autochthonous transmission of both dengue and chikungunya reported in southern France in 2010 and 2014,^93^, ^94^ local dengue transmission in Croatia in 2010,^95^ the largest dengue outbreak reported in China in 2014,^96^ and the first local dengue case reported in Japan in over 70 years in 2014.^97^ Chikungunya is also on the rise, with an outbreak reported in Italy in 2007^98^ and 2017, the first local transmission of this disease in the United States in 2014,^99^ and recent epidemics in the Caribbean, and Central and South American regions.^100^

*Ae. aegypti* is considered to be the most efficient urban vector of dengue in tropical and subtropical settings, while *Ae. albopictus* is more adapted to temperate climate regions (e.g., United States, Europe, Japan, and parts of China) and is more efficient at transmitting chikungunya virus.^101^, ^102^, ^103^ There is increasing evidence that (1) recent climate change has already favored *Ae. albopictus* to settling in temperate regions once it has been introduced, due to favorable overwintering and annual temperature conditions; (2) the mosquito has not yet filled its potential ecological niche; and (3) future climate change might sustain its establishment at higher latitudes in temperate regions.^104^, ^105^, ^106^, ^107^, ^108^, ^109^, ^110^, ^111^, ^112^, ^113^, ^114^ The current distribution of *Ae. aegypti* is more restricted to the tropics and subtropics,^78^, ^115^ and future scenarios suggest a moderate latitudinal shift in its potential ecological niche because its eggs do not tolerate temperate winters.^103^, ^113^, ^114^ However, it is noteworthy that *Ae. aegypti* used to be present around the Mediterranean basin and was reported as far north as Brest in France and Odessa in Ukraine after World War II;^116^ consequently, these scenarios might be slightly optimistic.

Several mechanistic and statistical disease modeling approaches have been employed to project the future distribution of dengue.^35^, ^113^, ^117^, ^118^, ^119^, ^120^ These studies generally project an increase in the overall burden of dengue, but a clear consensus is lacking regarding regions of the world where transmission is expected to intensify and expand or contract and diminish.^101^ A recent study highlights that future climate conditions might become increasingly suitable for dengue transmission in southern Europe in the summer.^121^ Recent projections carried out for chikungunya show increasingly suitable climatic conditions in temperate regions of Western Europe (France, the Benelux Union, and Germany) for the future.^122^, ^123^ The expected increase in urbanization (especially in urban slums) and increasing trends in international trade and travel must be expected to amplify, rather than reduce, future temperature effects on these diseases.^31^

In 2015, an outbreak of ZIKV hit Brazil before spreading to most countries in South and Central America and the Caribbean. In subsequent years, ZIKV circulated in a few countries in Africa and Southeast Asia. In summer 2017, limited and local transmission occurred in Texas and Florida in the southern United States. The virus was very likely introduced into northeastern Brazil by a traveler in 2014; the first human case was subsequently detected in May 2015.^124^ ZIKV is primarily transmitted by the bites of infected *Aedes* mosquitoes,^125^ and it can also be sexually transmitted. It causes severe neurological complications such as microcephaly in unborn children,^126^ and less frequently, a paralytic autoimmune disease called Guillain–Barré syndrome. WHO declared ZIKV as a Public Health Emergency of International Concern in February 2016. The end of this emergency was declared in November 2016 when the number of infected cases significantly declined.

When the outbreak started, scientists emphasized that the “2015 El Niño caused exceptional climatic conditions in north‐eastern South America during winter and spring in the Southern Hemisphere.”^127^ This statement was in agreement with previous studies demonstrating a significant link between the El Niño climate phenomenon, regional climate anomalies, and dengue epidemics in South America and Southeast Asia.^128^, ^129^ The positive phase of the El Niño Southern Oscillation has been associated with many infectious disease outbreaks worldwide, including Rift Valley fever, malaria, and cholera in East Africa; increased risk of arbovirus and malaria transmission in Latin America and Southeast Asia; and outbreaks of malaria and cholera in India, and this is just the tip of the iceberg.^130^, ^131^ El Niño is a natural climatic oscillation; however, it now occurs with a warmer sea surface temperature background and this poses serious health implications for the future, in particular in the tropical belt.^132^ A rainfall‐ and temperature‐driven model of the disease basic reproduction ratio (*R*_0_, the number of secondary infections produced by a single case introduced into a completely susceptible population) for ZIKV later confirmed that climatic conditions related to El Niño 2015–16 were optimal for the mosquito‐borne transmission risk of ZIKV in Latin America.^133^ The simulated transmission risk of ZIKV in South America in 2015 was the largest since the 1950s. Risk maps also reveal potential transmission risk in the southern states of the United States, the southern provinces of China, where dengue transmission has already been observed, and to a lesser extent in southern Europe. This work also predicted the spread of ZIKV to Angola; this country was plagued by yellow fever during the Latin American outbreak. ZIKV was found circulating in Angola in subsequent years.^134^ Recent modeling work corroborates these findings at the city‐scale; namely that climatic conditions were optimal for ZIKV transmission in Feira de Santana, Brazil in 2015–16.^135^ Another study highlights the worsening additional effect of an earthquake that hit Ecuador in 2016.^136^ Importantly, Muñoz and colleagues utilized a similar *R*_0_ model driven by operational seasonal climate forecasts to show that the ZIKV epidemic could have been theoretically forecast one month in advance in Brazil in 2015.^137^

Many other factors synergistically contributed to the severity of the ZIKV epidemic in Latin America in 2015–16. South American and Caribbean populations were very likely fully susceptible to infection before the virus was introduced.^138^ Additionally, storage of water in containers during droughts in urban slums combined with the anthropophilic behavior of *Ae. aegypti*, political instabilities, human behavior, and other natural disasters very likely led to the epidemic.^139^ It is very difficult to develop future scenarios for ZIKV risk, given that the level of herd immunity following the large‐scale exposure of human populations should prevent a large outbreak occurring in South America within the upcoming decade.^138^ However, semitropical and temperate regions where populations have never been exposed to ZIKV and where competent *Aedes* vectors and suitable environmental conditions co‐occur should be stringently surveyed by public health services.

Yellow fever has also re‐emerged in the Democratic Republic of the Congo and Angola in 2015–16 and in Brazil in 2017–18. Theoretically, yellow fever should not be a problem, given the availability of an effective vaccine that provides lifelong immunity to infection. However, the recent epidemics in Africa and Brazil, combined with the limited number of vaccine manufacturers approved by WHO, significantly depleted the global stock of available vaccine. Surveillance should concentrate on urban areas and slums where *Ae. aegypti*, the anthropophilic yellow fever mosquito, is present.

West Nile Virus (WNV), which infects birds, humans, horses, and other mammals, is the most widely distributed encephalitic flavivirus. WNV is mainly transmitted by *Culex* mosquitoes in all continents, except Antarctica. Many studies have already discussed the importance of weather and climate in driving WNV epidemics.^140^, ^141^, ^142^, ^143^ WNV has circulated in Africa since the 1930s and first appeared in New York City in the United States in 1999. WNV rapidly spread to four northeastern U.S. states, finally reaching California during summer 2003.^144^ Circulation in humans and horses was reported in the western Mediterranean region and southern Russia in the early 1960s, Belarus and Ukraine in the 1970s and 1980s, and a large WNV outbreak was reported in 1996–97 in Romania, followed by another epidemic wave in Russia in 1999.^145^ Large WNV epidemics were subsequently reported in Russia during the late 2000s^146^ and in the Balkan area in the early 2010s. Milder winter conditions, combined with droughts during the boreal spring season, were associated with increased risk of WNV transmission by urban mosquitoes in the United States.^141^ Extreme rainfall events were also associated with increased risk of WNV transmission. Given that the extrinsic incubation period of WNV in *Culex* mosquitoes shortens significantly when temperature increases,^14^ climate change will undoubtedly impact future WNV epidemics.

### Tick‐borne diseases

*Ixodes* ticks can transmit bacteria from the genus *Borrelia* (causing Lyme disease) and *Flaviviridae* virus (such as tick‐borne encephalitis, TBE). Lyme disease is the most common VBD affecting humans in the temperate Northern Hemisphere. Rainfall, moisture, and temperature affect the life cycle and habitat of *Ixodes* ticks, and they prefer habitats with at least 85% relative humidity and search for hosts when the temperature exceeds about 7 °C.^147^

*Ixodes ricinus*, the sheep tick, has expanded its geographical range and seasonal activity in Europe over the past decade,^148^ including its distribution, shifting farther north in Sweden and Norway. This northern shift and increase in activity is related to milder winters and prolonged spring and autumn seasons in the 1990s, combined with increased vegetative cover and the spread of deer carrying ticks into newly suitable regions.^149^ Similar trends have been observed in the Baltic countries and northern parts of Poland. In the Alps and the Carpathian mountains of central Europe, an altitudinal shift of *I. ricinus* from about 700 m in the 1950s to 1200 m in the 2000s has also been reported by field entomologists.^150^, ^151^, ^152^ Future scenarios indicate a potential further expansion of *I. ricinus* in northern and eastern Europe.^153^

The number of Lyme disease cases in Europe has steadily increased from about 3000 in the early 1990s to 35,000 in the late 2000s.^154^ In the United States, similar trends have been observed. Two main tick species, *Ixodes scapularis*, the deer tick on the east coast, and *Ixodes pacificus*, on the west coast, transmit Lyme disease in the United States; they both spread farther north between 1996 and 2015.^155^ The number of Lyme disease cases has more than doubled in the United States since the 1990s, and Lyme disease is now believed to affect about 300,000 Americans annually. Ogden and others have warned about the potential northern expansion of *I. scapularis* and the diseases it transmits into Canada for more than a decade.^156^, ^157^ The first infected ticks were found on the Ontario shore of Lake Erie in the early 1990s. *Ixodes* ticks have since spread farther north into Ontario, parts of Quebec, Manitoba, New Brunswick, and Nova Scotia.^158^ The incidence of Lyme disease has increased from 0.4 to 2.7 per 100,000 population from 2009 to 2016 in Canada; 88% of cases were reported in the provinces of Quebec, Ontario, and Nova Scotia.^159^ Even under an optimistic climate change scenario, for which the global warming increase is limited to 1.5 °C, consistent with the Paris agreement target, Lyme disease was found in simulations to spread farther north in Canada in the future.^160^ Northern Russia has also experienced an increase in the *Ixodes* tick population and in TBE cases over the past decades.^161^, ^162^ In particular, a 50‐fold rise in TBE incidence was reported for the far northern province of Arkhangelsk Oblast during the 2000s compared with the 1980s. There was also a distinct correlation between TBE incidence and increases in mean annual air temperatures from 1990 to 2009.^162^

Other important tick‐borne pathogens (babesiosis, Crimean‐Congo hemorrhagic fever, and rickettsioses) are also climate‐sensitive (for more detailed reviews, see Refs. 148 and 163). The patterns described for tick‐borne diseases, including an altitudinal and latitudinal shift of *Ixodes* ticks and the diseases they transmit, have been observed in different temperate and peri‐Arctic regions of the Northern Hemisphere during the past decade. This is related to the direct impact of climate change on *Ixodes* tick habitats, with more conducive temperature conditions and milder winters. Similar trends are likely for these regions in the future if we fail to mitigate and adapt to climate change. However, this is not the only factor causing tick spread into new habitats; other anthropogenic and natural factors need to be considered. Wild animal hosts (deer, other cervids, birds, and rodents) also carry tick vectors into new regions, and afforestation and land and wildlife management are extremely important potential drivers.^148^, ^164^ The expansion of towns and urban areas into green zones, combined with an increasing numbers of trekkers and wildlife lovers, is also changing human and domestic animal exposure to tick‐borne pathogens.

### Midge‐borne diseases: the example of bluetongue emergence in northern Europe

Bluetongue is a noncontagious, midge‐borne viral disease affecting ruminants (mainly sheep and less frequently cattle, goats, antelope, deer, camel, and dromedaries). Bluetongue virus (BTV) is transmitted by *Culicoides* biting midges. Its emergence in northern Europe in 2006 is considered a prime example of early climate change impacts on VBD.^165^ The Afrotropical midge vector *Culicoides imicola* was responsible for a large BTV outbreak in southern Europe in 1998.^166^ At that time, it was not expected that indigenous *Culicoides* midges (*Culicoides obsoletus*) could transmit BTV in the cold climate of northern Europe and Scandinavia. But in 2006, BTV emerged in northern European farms. From 2006 to 2009, tens of thousands of farms were unexpectedly affected by BTV. This outbreak resulted in a huge financial cost to the European farming industry: about €164–175 million in the Netherlands alone in 2007.^167^ It was brought to an end by a newly developed vaccine. However, in 2015, BTV re‐emerged in central and northern France, and has been present ever since, with livestock movements currently restricted country‐wide in an attempt to contain the area affected.^168^

Modeling studies have highlighted that climate conditions favored the spread of the Afrotropical midge vector in southern Europe as well as increasing the vectorial capacity of indigenous *Culicoides* vectors to transmit BTV in northern Europe in 2006.^169^ This outbreak coincided with the heatwave that hit Europe during summer 2006 (and caused a large increase in mortality in the elderly human population). Furthermore, the wind dispersal of infected midges and the movement of infected animals are important parameters to consider.^170^, ^171^ Future scenarios indicate that rainfall and temperature conditions will become increasingly suitable for the transmission of BTV in northern Europe in the far future.^169^

### Helminths and other parasites

In veterinary parasitology, the work of Ollerenshaw and Rollands on *Fasciola hepatica*, the sheep liver fluke, in Wales is a prime example of the impact of weather and climate on the dynamics of parasitic worms. The parasite develops on wet grassland, and then infects an intermediate snail host, before being ingested by a ruminant. *F. hepatica* then migrates to the bile duct and liver of the host, before releasing eggs which are excreted by the animal onto pasture. The free‐living stages of the parasite and the snails themselves are favored by wet and mild/warm climatic conditions on grassland.^172^ Transmission of the parasite in most countries is seasonal; it takes approximately three months for parasite stages to develop from eggs and for the release of infective metacercariae onto pasture to occur via the intermediate snail host.^172^ Liver fluke causes severe morbidity in sheep, reduced milk yield in dairy cattle, reduced growth rates, and liver condemnation; consequently, it has large cost implications for the farming industry. Ollerenshaw and Rollands developed an empirical model to forecast the risk of acute fluke infections in sheep during the late 1950s. This model relies on the frequency of rainy days, evapotranspiration, and temperature from spring to early fall to forecast acute disease burden in sheep the following winter; the model takes advantage of the time lag associated with the life cycle of the parasite. The model was able to successfully forecast epidemics of liver fluke in sheep in Anglesey, Wales in the late 1950s. The model is now used operationally by the National Animal Disease Information Service to provide regional risk forecasts to UK farmers.^173^ Other spatial risk models based on evapotranspiration data from satellite have been subsequently developed to model the risk posed by liver fluke.^174^

Recent studies have shown that climatic conditions are increasingly suitable for the transmission of liver fluke in the UK,^175^ some temperate northern European regions,^176^ and New Zealand.^177^ Interestingly, the number of ruminants infected by liver fluke and other helminths significantly increased during the 2000s in the UK^178^ and in some European regions. This increase might have been caused by the combination of increasingly suitable environmental conditions combined with an increase in anthelminthic drug resistance.^179^ Future scenarios suggest a lengthening and intensification of the liver fluke transmission season in northern Europe.^176^ Under the most extreme greenhouse gas emission scenario (RCP8.5), fluke burden in Scotland in the 2080s might become as large as currently reported for the most endemic region in the UK, West Wales, and the Valleys. Similar trends are likely for the barber pole worm, *Haemonchus contortus*, in Europe.^180^ Other climate‐sensitive helminths will also be significantly affected by climate change in temperate regions.^178^

Schistosomiasis is caused by five species of the flat worm *Schistosoma* and requires aquatic snails as intermediate hosts to complete its life cycle. Its global prevalence has increased since the 1950s largely as a result of expansion of irrigation systems in hot climates, where the snail host and parasite can infect human hosts. However, the recent scaling up of distribution of the drug praziquantel to school children and adults in Africa, where most cases occur, has significantly decreased disease prevalence. The parasite and aquatic snail require specific water temperature conditions. In 2015, the first local case of schistosomiasis was reported in Corsica, France.^181^ The availability of competent snail hosts, combined with warm summer temperature conditions, and the importation of the parasite by an infected traveler led to the infection of several family members who were swimming in a river. Climate change is expected to significantly increase infection risk in East Africa by 20% over the next 20–50 years,^182^ and it might spread farther north into nonendemic areas of China by the 2050s.^183^

Kutz and others have extensively studied the ecology of host–parasite interactions in the Arctic environment.^184^, ^185^, ^186^, ^187^ The Artic provides interesting insights into potential changes in future host–parasite systems. Helminth parasites (the lungworm *Umingmakstrongylus pallikuukensis* and *Varestrongylus eleguneniensis*) affecting muskoxen and caribou and their gastropod intermediate hosts have rapidly extended their range at high latitudes in the central Canadian Arctic. These parasites were found in the late 2000s on Victoria Island. Their spread perfectly coincided with accelerated warming across the region. Cool climatic conditions before the 2000s may have restricted establishment of lungworms and intermediate hosts on the island. The spread of these parasites by migratory caribou is very likely, and this was further facilitated by rapidly changing environmental conditions. This is also consistent with faster warming occurring at high latitudes and altitudes; unfortunately, climate models show that similar trends are likely in future. Furthermore, muskoxen were severely hit by the direct impacts of climate change on their environment in 2018.^202^ Rising temperatures now favor rainfall instead of snowfall at high latitudes. Once rainfall reaches the surface, it rapidly freezes, encasing the plants in ice and making them inaccessible to the animals that feed on them. Similar trends have been reported in the Southern Hemisphere, in East Antarctica, where a colony of about 40,000 Adélie penguins died from starvation in 2017. More recently, in 2016, an outbreak of anthrax, a bacteria present in soil, affected reindeer en masse in Siberia. Anthrax spores can survive in frozen animal and human cadavers for hundreds of years. Following the 2016 reindeer outbreak, 72 nomadic herders, including 41 children, were hospitalized due to subsequent infection by the bacteria, also known as Siberian plague; one child died. Experts related this catastrophic event to unusually warm weather conditions; the Russian authorities subsequently culled the reindeer population to limit further spread of anthrax.

### Water‐borne diseases

*Vibrio cholerae*, the bacteria that causes cholera, is naturally present in the environment, in particular in coastal and estuarine ecosystems. Rita Colwell devoted her life to studying *Vibrio* bacteria and their ecosystem. *V. cholera*, like other species within the genus, attaches to copepods; the survival of these bacteria and their multiplication consequently rely on plankton population density and environmental variables such as sea surface temperatures and the input of fresh water.^188^ Floods combined with abnormally warm sea surface temperatures favor cholera outbreaks.^189^ An early warning system based on climatic variables was subsequently developed to assess the risk posed by cholera in Chesapeake Bay, United States. Critically, the quality of public health services, access to clean water and sanitation, and political stability are basic factors impacting cholera outbreaks; a prime example is the recent epidemic in Yemen.^190^ The emergence of many other water‐borne diseases is climate sensitive^6^, ^191^ and strongly associated with increases in rainfall extremes and hurricanes. For example, a large cryptosporidium outbreak followed floods from the Mississippi in Milwaukee in 1993, and various toxins and norovirus spread in Katrina's wake in Louisiana in 2005.^192^

## Recent methodological progress and research relevance to decision makers

Arguably, the most significant challenge in VBD risk modeling is the diverse nature, quality, and accessibility of observational datasets, particularly in the low‐income countries which bear the largest burden of VBDs. This, coupled with the complex, multilayer, multiscale, dynamic nature of the disease system, has resulted in a huge variety of VBD models, ranging from global‐scale statistical models of risk to agent‐based models that simulate VBD infection within human hosts at the individual scale.

To date, assessments of the impact of climate change on future VBD risk have primarily been carried out at continental to global scales, either using statistical models linking vector or pathogen presence to environmental and socioeconomic factors, or by using a mechanistic approach such as calculating the disease basic reproduction ratio, *R*_0_, using SEIR/SIR types of models based on differential equations or using degree–day types of models. The two approaches each have advantages and disadvantages.^193^, ^194^ While statistical models are derived directly from observations and may employ statistical fitting methods which inherently estimate their own uncertainty, they generally tend to produce static maps and do not capture the dynamic relationship between climate and VBD risk, which usually follows a seasonal pattern. Furthermore, statistical models cannot extrapolate risk to different scenarios of intervention or climate patterns beyond those within the training data set. Mechanistic models, on the other hand, have the advantage of explicitly modeling the climate–disease risk relationship, but need detailed information about vector and pathogen processes to parameterize the model, and these are often obtained from a collection of small‐scale studies in the laboratory or field. Additionally, some mechanistic model parameters rely on laboratory experiments carried out in the 1950s–1960s, and these must be updated for different vector–pathogen combinations.^195^ On a positive note, this work is in progress. Mechanistic models also require validation of their simulated risk against observational data. Long time series are needed to robustly assess the predictive skill of such models. When verified as being able to reproduce past outbreaks, such models can then be used with seasonal climate forecasts (for a detailed review, see Ref. 196) or climate change projections to develop scenarios describing future risk.

It should be noted that mechanistic models based on *R*_0_ have their own limitations due to simplification; while useful for large‐scale risk assessment, they do not resolve small spatial and temporal scale variability, and make simplifying assumptions regarding homogeneity of the hosts, vectors, and their interactions. Capturing the heterogeneity in lower transmission settings has been identified as a key modeling challenge for malaria,^197^ and such settings are often also those most likely to be sensitive to climate. Furthermore, while mechanistic *R*_0_ models predict only the suitability of climate conditions for vector or disease transmission if they are introduced, they do not estimate the probability of occurrence, which depends on a dispersal route from endemic regions to unaffected areas.

Recently, some researchers have begun to combine mechanistic and statistical modeling approaches to derive improved VBD risk assessments.^198^, ^199^ Such methods maybe key in solving the technical challenges of combining accurate models with diverse datasets to provide informative risk predictions. High‐resolution risk maps based on statistical models are very useful resources to inform control measures in countries plagued by VBD and estimate spatial patterns of drug and insecticide resistance,^200^ a requirement to meet the demand for locally adapted vector control. But temporal variability is also important; as Bill Gates recently said, “Malaria isn't just patchy. It's also spiky. We need to know when those spikes happen, so that we can mobilize.” Mechanistic VBD models relying on rainfall and temperature can reproduce spikes of past VBD outbreaks at broad spatial scales (examples include the bluetongue outbreak in Northern Europe in 2006, ZIKV epidemic in South America in 2015, and historical dengue outbreaks in Mexico and Nicaragua). Consequently, these need to be tested operationally in collaboration with governmental agencies, decision makers, and public health services, as is currently happening.^137^ Importantly, both correlative and mechanistic modeling approaches have correctly anticipated the spread of important vectors worldwide; *Ae. albopictus* in Europe and *I. scapularis* in North America are key examples.

Critically, the interrelatedness of the impacts of climate change on economics and VBD control measures needs to be investigated further. There is also a need for long‐term monitoring of vector activity and the diseases they transmit in order to better detect and attribute effects of climate change on VBD emergence and re‐emergence. This can be achieved by funding health observatories and improving disease surveillance systems worldwide.

Significant progress has been achieved in terms of future risk assessment of VBDs over the past decade. An increasing number of studies now utilize an ensemble of climate models driven by different greenhouse gas emission scenarios, coupled with different population scenarios to drive future health impact models. This is critical in order to estimate and communicate the related uncertainties, which can be quite large. Climate model outputs have to be calibrated with respect to climate observations, as they still suffer from systematic biases. Stringent disease model validation over the past is a prerequisite for providing future projections. As climate models have benefited from the development of earth observation systems (satellites and weather stations), disease model validations will increasingly benefit from an improvement in disease surveillance systems worldwide (e.g., the gold standard Global Burden of Disease project). Health impact model intercomparison projects should also be encouraged. The climate community has been comparing climate model outputs produced by different climate centers for many years now (for the Coupled Model InterComparison Project). The impact modeling community recently started to do so, for key sectors such as agriculture, water resources, and health.^201^ Multidisciplinary research and collaboration between health professionals, epidemiologists, statisticians, and computer and earth scientists will be key to anticipating and addressing the health challenges to come.

## Conclusion

There is a wealth of evidence that recent climate change has already affected pathogen–vector–host systems, in particular over temperate, peri‐Arctic and Artic areas and high altitude regions in the tropics. There are now many examples of the early impacts of climate change on animal VBD burden, while the most severe VBD outbreaks affecting humans tend to be affected by a myriad of complex socioeconomic factors and climate. Our review demonstrates that the spread of vectors and the pathogens they transmit worldwide has been anticipated by scientists. Similar trends are likely in the future if humans fail to mitigate and adapt to climate change and if drug and insecticide resistance continue to rise. On a positive note, significant progress has been achieved in terms of surveillance systems, disease and vector control measures, vaccine development, diagnostic tests, and mathematical risk modeling/mapping in recent decades, thanks to significant funding efforts from governments, non‐governmental organizations (e.g., charities), and patrons from within the private sector (e.g., the Bill and Melinda Gates Foundation). Critically, research funding efforts and public health infrastructures need to be sustained if we do not want to repeat the past.
